# Development and Percutaneous Permeation Study of Escinosomes, Escin-Based Nanovesicles Loaded with Berberine Chloride

**DOI:** 10.3390/pharmaceutics11120682

**Published:** 2019-12-15

**Authors:** Giulia Vanti, Daniele Bani, Maria Cristina Salvatici, Maria Camilla Bergonzi, Anna Rita Bilia

**Affiliations:** 1 Department of Chemistry, University of Florence, Via Ugo Schiff 6, 50019 Sesto Fiorentino (FI), Italy; giulia.vanti@unifi.it (G.V.); mc.bergonzi@unifi.it (M.C.B.); 2 Department of Experimental and Clinical Medicine, University of Florence, Viale Pieraccini 6, 50139 Firenze, Italy; daniele.bani@unifi.it; 3 Centro di Microscopie Elettroniche “Laura Bonzi” (Ce.M.E.), ICCOM, National Research Council (CNR), Via Madonna del Piano 10, 50019 Sesto Fiorentino (FI), Italy; salvatici@ceme.fi.cnr.it

**Keywords:** vesicles, berberine chloride, escin, dermatological use, rabbit ear skin, human abdominal skin

## Abstract

Escin is a natural saponin, clinically used for the anti-edematous and anti-inflammatory effects. The aim of the study was to explore the possibility of converting escin into vesicle bilayer-forming component. The hyaluronidase inhibition activity of escin was evaluated after its formulation in escinosomes. Berberine chloride, a natural quaternary isoquinoline alkaloid isolated from several medicinal plants that is traditionally used for various skin conditions was loaded in the vesicles. The developed nanovesicles were characterized in terms of diameter, polydispersity, ζ-potential, deformability, recovery, encapsulation efficiency, stability, and release kinetics. Nanovesicle permeation properties through artificial membranes and rabbit ear skin were investigated using skin-PAMPA^TM^ and Franz cells were also evaluated. Escinosomes, made of phosphatidylcholine and escin, were loaded with berberine chloride. These nanovesicles displayed the best characteristics for skin application, particularly optimal polydispersity (0.17) and deformability, high negative ζ-potential value, great encapsulation efficiency (about 67%), high stability, and the best release properties of berberine chloride (about 75% after 24 h). In conclusion, escinosomes seem to be new vesicular carriers, capable to maintain escin properties such as hyaluronidase inhibition activity, and able to load other active molecules such as berberine chloride, in order to enhance or expand the activity of the loaded drug.

## 1. Introduction

The majority of pathological skin conditions are treated with topical formulations delivering therapeutically effective concentration of drugs in skin layers and exert a local effect. By contrast, many potent drugs display scarce ability to penetrate the skin, in particular the *stratum corneum*, which represents the main obstacle that limit their absorption after topical application and hence inherent potency. Particularly, many skin inflammatory diseases are characterized by leucocyte invasion leading to a proliferation and abnormal differentiation of keratinocytes with a subsequent increase in skin thickness and alterations of skin barrier properties [1]. Nano-delivery systems can represent proper carriers for skin delivery of suitable therapeutic drug concentrations within the deeper skin layers and provide a sustained and/or controlled release [2]. Phospholipid-based nanovesicles are particularly suitable for topical formulations because of their high biocompatibility with skin lipids and negligible toxicity [3]. Conversely, conventional liposomes composed of pure or mixtures of phospholipid(s) and cholesterol generally accumulate in the *stratum corneum* and in the skin appendages, with negligible permeation through deeper skin layers, because of their low flexibility, as reported by most of the literature [4,5,6].

Some molecules can impart deformability or elasticity to the bilayer membrane of nanosized liposomes, resulting in markedly improved drug permeability through the skin by several orders of magnitude [7,8,9]. Besides yielding a deeper penetration through skin, nanovesicles can effectively protect the drug against the environment, retain the drug in the target tissue, reduce systemic absorption and minimize side effects [10].

**Figure 1:**
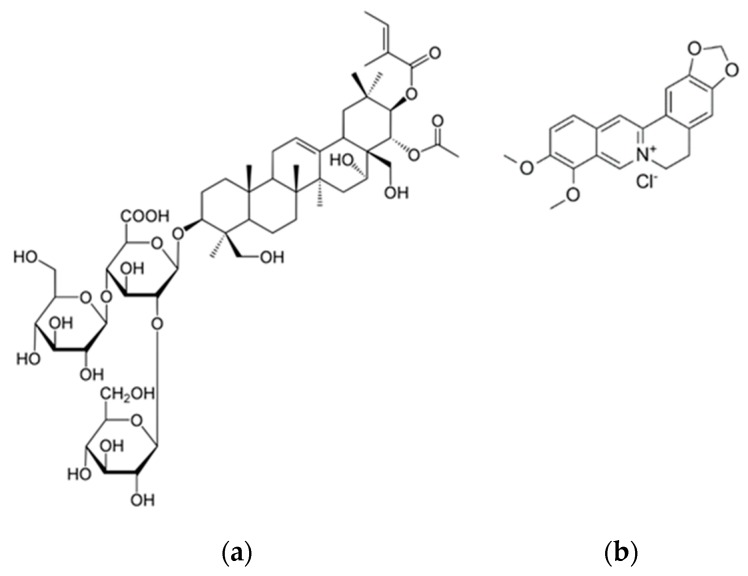
(**a**) ESN (escin) structure and (**b**) BRB (berberine chloride) structure.

The principal aim of the present work was to design and develop novel nanovesicles for topical delivery loaded with escin (ESN, Figure 1a), a natural bioactive saponin, and berberine chloride (BRB, Figure 1b), a quaternary isoquinoline alkaloid.

Natural products represent attractive molecules in drug discovery for their broad structure variability and also for their capability to interact with multiple targets, representing a more versatile approach to successful therapies [11,12,13]. Saponins are natural non-ionic surfactants with useful pharmacological and biological properties, mainly distributed in plants, lower marine animals, and some bacteria. Saponins contain an aglycone with a steroid (C27) structure or a triterpene (C30) moiety, represented by a four-ringed steroid nucleus or a five-ringed structure. The amphiphilic properties of saponins are due to sugar moieties attached with one (monodesmosidic saponins, usually glycosylated at C-3 position) or two (bidesmosidic) hydroxyl or carboxyl moieties. When dissolved in water, saponins produce foam, which is related to their name from the Latin word sapo [14].

ESN is the major active saponin isolated from the seed of *Aesculus hippocastanum*. ESN is a triterpenoid glycoside with three sugar units linked at C-3 of the aglycone moiety, and consisted of a mixture of α-escin and β-escin. ESN is clinically used to increase venous wall tone and to achieve antiedematous and anti-inflammatory effects, accounting for its efficacy in the treatment of chronic venous insufficiency, hemorrhoids, and post-operative oedema [15]. It has been shown that, after application on dorsal and ventral skin in mice and rats, 25% and 50%, respectively, of the topical dose was absorbed by the skin. Following topical administration in pigs, high ESN concentrations were found under the site of application, even in deeper muscle structures [15].

Previously, a study using 1,2-dimyristoyl-sn-glycero-phosphocholine (DMPC, 15 mg/mL) vesicles evaluated the effects of ESN addition. Vesicles were stable below 1 mol % of ESN. Between 1 and 6–7 mol % of ESN, the formation of vesicles was also observed, but large aggregates that precipitate over time occurred. Micelle-sized structures were formed at ESN contents above 6–7 mol % [16].

In a further study, the interaction of ESN with cholesterol and natural dipalmitoylphosphatidylcholine monolayers was investigated using Langmuir isotherms [17]. The monolayer was obtained by DPPC and cholesterol or a mixture of them. Intercalation of ESN in a monolayer made of pure cholesterol was low, whereas in a pure DPPC monolayer, no intercalation of ESN was detectable. By contrast, in aqueous pseudo-ternary systems (ESN, dipalmitoylphosphatidylcholine, cholesterol) and in pseudo-binary systems (ESN, cholesterol), colloidal microstructures built up from ring-like and worm-like subunits were observed [17].

BRB is a natural quaternary isoquinoline alkaloid isolated from several medicinal plants of the genera *Berberis*, *Hydrastis*, *Coptis*, *Coscinium*, and *Mahonia*, which are traditionally used for various skin conditions. BRB is a representative natural constituent having a variety of biological effects through different mechanisms of actions [18]. Despite the evidenced potentiality of BRB in the treatment of skin diseases, its topical application is limited due to its high hydrophilicity, the aqueous solubility of 1–2 mg/mL at 25 °C and the approximate log *p* value of −1.5 hinder its delivery across the skin layers [19]. Recently, to increase its dermal bioavailability, BRB was formulated with sodium oleate as complexing agent, because of its low toxicity and skin penetrating characteristics. This complex displayed about 250-fold higher saturation solubility in *n*-octanol, endorsing the improved lipid solubility of the complex compared with free drug [20].

The present study was designed to explore the conversion of the biologically active amphiphilic saponin ESN, into a vesicle bilayer-forming component. In order to assess that ESN potential activity remained after the formulation in the vesicles, the hyaluronidase inhibition activity of these escinosomes was evaluated. Nanovesicle deformability after vesicle loading with BRB was also examined, in order to evaluate their passage through the skin and BRB transport in the different skin layers. A further aim of the present work was to investigate the anatomical similarities of rabbit ear skin with human abdominal skin with respect to the *stratum corneum* and epidermal thickness as well as the hair follicular density, in order to evaluate permeation properties of the different formulations using vertical diffusion Franz cells.

## 2. Materials and Methods

### 2.1. Materials

Phospolipon 90G (P90G) was purchased from Lipoid AG (Cologne, Germany) with the support of its Italian agent AVG srl. Cholesterol (CHOL), ESN, BRB and hydroxypropyl methylcellulose (HPMC) were provided by Sigma-Aldrich (Milan, Italy). Methanol (MeOH), methanol HPLC grade, acetonitrile (ACN), formic acid, dichloromethane (CH_2_Cl_2_), dimethylsulfoxide (DMSO) and formaldehyde solution, phosphate saline buffer (PBS), acetate buffer, NaOH, potassium borate, hyaluronidase from bovine testes Type IV-S, powder (mouse embryo tested, 750–3000 units/mg solid), compound 48/80 (condensation product of *N*-methyl-*p*-methoxyphenethylamine with formaldehyde, hyaluronic acid potassium salt from human umbilical cord, *p*-dimethylaminobenzaldehyde (98%, Ehrlich′s reagent) were purchased from Sigma-Aldrich (Milan, Italy). Piroxicam, progesteron, Prisma Buffer (P/N 110151), Hydration Solution (P/N: 120706) and Skin-PAMPA^TM^ system were purchased from pION Inc. (Billerica, MA, USA). Ultrapure water was produced by a synergy UV Simplicity water purification system provided by Merck KGaA (Molsheim, France). Phosphotungstic acid (PTA) was purchased from Electron Microscopy Sciences (Hatfield, PA, USA).

### 2.2. HPLC-DAD Analytical Method

Quantitative determination of active constituents was carried out by the 1200 High Performance Liquid Chromatograph (HPLC) equipped with a Diode Array Detector (DAD) from Agilent Technologies Italia Spa (Rome, Italy). DAD was set up at the two wavelengths of 210 nm for ESN and 346 nm for BRB. Chromatographic analysis were performed using a reverse-phase column Luna-C18, 250 mm × 4.6 mm, 5 µm particle size, maintained at 27 °C. A gradient elution method with 1 mL/min flow rate for 32 min was applied, using (A) acetonitrile and (B) water at pH 3.2 (by formic acid) like mobile phases. Acetonitrile was selected because of its low UV cut off, which avoids interference with the ESN signal. The analytical method was: 0–3 min 85% (B), 3–9 min 85%–70% (B), 9–10 min 70% (B), 10–17 min 70%–50% (B), 17–19 min 50% (B), 19–26 min 50%–45% (B), 26–30 min 45%–40% (B), 30–35 min 40%–1% (B), 35–40 min 1%, 40–47 min 1%–85% (B).

R^2^ was calculated both for BRB and ESN and resulted respectively 0.99925 and 0.99979. Limit of detection (LOD) and limit of quantification (LOQ) were calculated both for BRB and ESN by determination of signal-to-noise ratio, in accordance to the ICH guidelines. LOD was 0.60 ng for BRB and 400.00 ng for ESN, while LOQ was 1.51 ng for BRB and 988.00 ng for ESN.

### 2.3. Development and Optimisation of Nanovesicles

Nanovesicles were prepared using the thin layer evaporation method at specific conditions [21,22]. Different liposomes were developed by varying the lipid constituents and loading BRB. In particular, 330 mg of P90G, 100 mg of CHOL and 50 mg of ESN were used as lipophilic components and they were dissolved in CH_2_Cl_2_/MeOH, while 13 mg of BRB were solubilized in water (10 mL). The organic solvent was removed by rotary evaporator for 20 min and the dried lipid film was then hydrated with the aqueous phase (with or without BRB) for 30 min at 35 °C [23]. First, conventional liposomes with P90G and CHOL were prepared; then, the same liposomes were prepared by adding ESN; finally, in the third formulation, P90G plus ESN were used to prepare the vesicles. The same three types of vesicles, loaded with BRB, were prepared in parallel. Finally, the Sonopuls Ultrasonic Homogenizer HD 2200 by Bandelin electronic GmbH & Co. KG (Berlin, German), was used in order to reduce vesicle size and improve sample homogeneity.

### 2.4. Physical Characterization of Nanovesicles

Nanovesicle characterization during the development and the optimization steps was made by measuring size, homogeneity and possible aggregation state by Dynamic and Electrophoretic Light Scattering (DLS and ELS, Zetasizer Nanoseries ZS90) by Malvern instrument (Worcestershire, UK), with a scattering angle of 90° at 25 °C [24,25]. Average Hydrodynamic Diameter (AHD, nm), size distribution expressed as Polydispersity Index (PdI, dimensionless measurement) and ζ-Potential (mV), were obtained using the software provided by Malvern. Scattering was measured on samples diluted 50/100-fold in ultrapure water and every measurement was performed in triplicate. Nanovesicle morphological characterization was performed by transmission electron microscope (CM12 TEM, PHILIPS, The Netherlands) equipped with an OLYMPUS Megaview G2 camera with an accelerating voltage of 80 kV. TEM allowed to visualize vesicle dispersion and dimensions. A drop of the diluted sample was applied and dried by desiccation on a carbon film copper grid. This was then counterstained with 1% (*w/v*) of phosphotungstic acid solution and examined at different magnifications [26]. The deformability of vesicles was measured by extrusion [27]. It was performed by 21 passages through polycarbonate extrusion membranes with 50 nm cut-off, using the LiposoFast-Basic extruder by Avestin Europe GmbH (Mannheim, Germany) joined to a 7 atm pressure source. Before the extrusion, each formulation was properly diluted in water. Deformability was expressed as the ratio among vesicles diameter before and after extrusion (Equation (1)). Values around the unit are index of good deformability.

Deformability = average diameter before extrusion/average diameter after extrusion,(1)

### 2.5. Technological Characterization of Nanovesicles

In order to characterize the liposomal dispersions, the encapsulation efficiency (EE) and the total recovery (R) were calculated for each formulation. EE is defined as the percentage of entrapped drug in relation to the weighed drug (Equation (2)):

EE% = encapsulated drug/weighted drug × 100%,(2)

In order to determine the amount of entrapped drug, vesicles were purified from free BRB and free ESN, by dialysis bag method [28,29], using Spectra/Por^®^ regenerated cellulose membranes with 3.5 KDa molecular weight cut-off (MWCO), by Repligen Europe B.V. (Breda, The Netherlands). The dialysis bag was stirred in ultrapure water (1 L), at room temperature for 1 h. After the purification, the samples were diluted in methanol, in order to dissolve and break the vesicles and release the encapsulated substances. Finally, a centrifugation at 14000 rpm for 10 min was applied and the amounts of BRB and ESN were determined by HPLC-DAD. Rather, R is defined as the percentage of total drug recovered after the preparation procedure in relation to the weighed drug (Equation (3)):

R% = total recovered drug/weighted drug × 100%,(3)

and it was measured using the same procedure without the purification step by dialysis. Moreover, the pH-meter (Basic 20+) by Crison Instrument (Barcelona, Spain) was used to measure the pH of each formulation.

### 2.6. Stability Studies

The stability studies were carried out storing the samples for 1 month at 4 °C in the dark [30]. Every ten days, physical (size, PdI, ζ-potential) and chemical (EE% and R%) parameters of the vesicles were investigated by DLS/ELS and HPLC-DAD.

### 2.7. Release of Active Constituents from the Vesicles

The dialysis bag method, with Spectra/Por^®^ regenerated cellulose membranes of 3.5 KDa MWCO by Repligen Europe B.V. (Breda, The Netherlands), was applied to study the release kinetic of ESN and BRB from liposomes [30,31] and from aqueous solution (BRB aqueous solution, B-SOL) or dispersion (ESN water dispersion, E-W), at the same concentrations used in liposomes, 1.3 mg/mL and 5 mg/mL respectively. 250 mL of PBS (pH 7.4) was used as release medium and the experiment was carried out in sink conditions at 37 °C under magnetic stirring, until 24 h. Therefore, 0.5 mL withdrawals were done at specified time points (30, 60, 120, 240, 360, and 1440) min and replaced by equal volumes of fresh buffer. Finally, all samples were centrifuged at 14,000 rpm for 10 min and analyzed by HPLC-DAD.

### 2.8. Hyaluronidase Inhibition Assay

The hyaluronidase inhibition activity was investigated using a colorimetric assay reported by Murata and coworkers [32]. Samples were dissolved in 0.1 M acetate buffer (pH 4.0, 0.2 mL) and were mixed with 0.1 mL hyaluronidase (400 unit/mL buffer) and incubated for 20 min at 37 °C. Next, compound 48/80 in buffer (0.3 mg/mL, 0.2 mL) was added as activator and allowed to react for 20 min at 37 °C. Then, hyaluronic acid potassium salt in buffer (0.4 mg/mL, 0.5 mL) was added and the mixture was incubated for 40 min at 37 °C. NaOH (0.4 M, 0.2 mL) was added to the mixture and cooling with ice stopping the reaction. Then, potassium borate solution (0.4 M, 0.2 mL) was added, vortexed and the mixture boiled for three minutes. The mixture was cooled using ice and 6 mL of a solution of p-dimethylaminobenzaldehyde (1% *w/v* in acetate buffer) was then added and the sample was placed for 20 min at 37 °C. The hyaluronidase inhibitory activities (HIA%) were measured at 600 nm, mean ± SD (*n* = 3), and calculated as follows:

HIA% = [(control − control blank) − (sample − sample blank)]/(control − control blank) × 100%,(4)

where control was acetate buffer and blank was the hyaluronidase enzyme in acetate buffer.

### 2.9. Skin-PAMPA™: In Vitro Simulation of Stratum Corneum Permeation by Vesiscles

The Skin-PAMPA^TM^ model from pION was employed as in vitro permeation assay, in order to evaluate and compare the permeability of the three formulations with BRB and predict BRB skin absorption [33]. B-SOL, was used as reference of free-BRB permeability. The skin-PAMPA membranes were used after an overnight hydration by the Hydration Solution, until all wells turned into translucent. After that, the wells in the bottom plate (donor) were filled with 200 µL of samples, while the wells in skin-PAMPA^TM^ top plate (P/N: 120656, pION) (acceptor) were filled with 200 µL of fresh acceptor solution, Prisma Buffer, at pH 7.4. A lid was used to cover the top plate, once the sandwich was assembled and a Parafilm layer was applied around the perimeter to seal the two compartments. Then, the 96-well STIRWELL^TM^ PAMPA sandwiches (P/N: 110243, pION) were incubated into a chamber with a wet filter paper under the lid, to maintain a high relative humidity and minimize evaporation. The assay was carried out at room temperature (25 °C ± 2 °C) for 5 h, according to the protocol. After the permeation time had elapsed, samples from acceptor and donor plate were collected and analyzed by HPLC-DAD. The Effective Permeability (P_e_) expressed as cm/s was calculated according to the following approximate equation [34]:

P_e_ ≈ (−2.303 V_D_)/(A t) log_10_ (1/(1 − R) C_D(t)_/C_D(0)_), (5)

where *A* is the filter area [0.3 cm^2^], multiplied by a nominal porosity of 70% according to the manufacturer, *V_D_* is the donor volume [0.2 cm^3^], *t* is the incubation time, *C*_D(t)_ is the concentration [mol x cm^−3^] of the compound in the donor phase at time *t*, *C*_D(0)_ is the initial concentration [mol × cm^−3^] of the compound in the donor phase at time 0, and *R* is the membrane retention factor (Equation (5)):

R = 1 – C_D(t)_/C_D(0)_ – V_A_/V_D_ ∙ C_A(t)_/C_D(0)_, (6)

and *r_a_* is the sink asymmetry ratio (gradient-pH-induced), defined according to the following equation:

r_a_ = V_D_/V_A_ ∙ P_e (A__→D)_ / P_e (D__→A)_, (7)

### 2.10. Histological Evaluation of Rabbit Ear Skin and Human Abdominal Skin

Cross-sections of rabbit ear skin were prepared at the Department of Experimental and Clinical Medicine, of the University of Florence, Florence, Italy. Human skin samples were kindly provided by operators from the Surgical Clinic, Careggi General Hospital, Florence, Italy, taken from surgical specimens removed for reductive abdominoplasty and intended for disposal. Both the rabbit and human skin samples were taken from discarded specimens intended for disposal, which exempted us from the need for a preliminary ethical permission. In the present study, the histological comparison between human abdominal skin and rabbit ear skin was investigated by conventional light microscopical analysis, in order to confirm the suitability of this animal model in the percutaneous studies [35]. Whole-thickness rabbit skin tissue was peeled off the inner part of the ear, close to the acoustic meatus, since preliminary studies showed little or no permeation through the skin from the ear apex. In particular, skin layers were manually stripped from the underlying cartilage within a rectangular incision made with a sharp blade and cut into discs 2 cm in diameter, paying attention not to damage the skin surface. Skin integrity was verified by light microscopy.

### 2.11. Percutaneous Penetration Studies using Rabbit Ear Skin

The skin discs thus prepared were used for the permeation study by the Franz cell system [36], in order to compare the permeation characteristics of the three formulations with BRB and BRB aqueous solution; all of them having the same BRB concentration (1.3 mg/mL). In order to avoid dehydration, the freshly cut skin was kept moisten between sheets of filter paper wet with PBS and the skin outer surface was gently dried by dabbing with filter paper, before placement on the vertical diffusion Franz cells (3.14 cm^2^ diffusion area). The donor chamber was filled with 1 mL of sample and the acceptor chamber was filled with 7 mL of PBS under magnetic stirring. During the experiment, the apparatus was maintained at 37 °C by a thermostatic bath circulation. The skin was placed between the two compartments, with the external layer facing the donor chamber and with dermis touching the buffer solution in the acceptor chamber. A suitable O-ring seal was used between the donor chamber and the skin, in order to prevent leakage of the sample and its spreading out over the entire skin surface [37]. Finally, the cells were clamped together and the donor chamber was sealed by Parafilm to avoid evaporation during the assay. The system was incubated for 6 h and 24 h. After 24 h, the acceptor solutions were analyzed by HPLC-DAD after 14,000 rpm centrifugation for 10 min, while the donor samples were first diluted with MeOH in order to allow vesicle break and fully dissolution of ESN/BRB. The amounts of BRB and ESN retained in the skin were also determined. Each skin lamina was washed 3 times with 3 mL PBS and dried with filter paper. The inner part of the skin specimen, in contact with the sample, was cut and divided into equal pieces. These were soaked in 1 mL of methanol, exposed to 3 h of ultrasonication bath and then shaken in a water bath for 3 h, in order to extract all the accumulated drugs [38]. Finally, the samples were centrifuged 10 min at 14,000 rpm and analyzed by the liquid chromatograph. The permeation parameters were calculated as following: absorbed dose (A_24_), expressed as percentage of the applied dose absorbed through the skin and that recovered in the receptor compartment; absorbable dose retained inside the skin (S_24_), expressed as percentage of the applied dose recovered inside the skin; total absorbed dose (TA_24_), expressed as sum of the two percentages A_24_ and S_24_.

### 2.12. Histological Studies of Rabbit Ear Skin after Percutaneous Penetration Studies

After the permeation experiments, 6 h and 24 h respectively, fragments of cutaneous tissue were resected from the central part of each skin disc exposed to the different BRB samples, fixed in cold 4% formaldehyde in 0.1 M PBS, pH 7.4, at 4 °C for 4 h, cryoprotected by 5 min incubation in cold saccharose 10% in PBS, washed in PBS, embedded in KillikTM medium by Bio-Optica (Milan, Italy), quickly frozen by immersion in isopenthane and stored at −80 °C until needed. Sections, 7 µm thick, were cut with a Reichert/Leica 2800 Frigocut cryostat (Wetzlar, Germany). Part of specimens were stained with hematoxylin and eosin (HE) for conventional light microscopical analysis, others were observed directly under a Zeiss Axioskop fluorescence microscope (Oberkochen, Germany) equipped with a λ 350 filter, close to the absorption peak of BRB, which emits in the yellow-green region of the visible spectrum (λ 550). The fluorescence of BRB was assumed as an index of drug penetration into different skin tissues.

## 3. Results

### 3.1. Development and Optimisation of Nanovesicles

Three different nanovesicles were developed using the thin layer evaporation method. Firstly, CL were formulated using P90G and CHOL using, respectively, 33 and 10 mg/mL of formulation. Subsequently, ESN (5mg/mL) was added to form conventional liposomes loaded with ESN (ECL). Finally, a third type of vesicle was formulated using only ESN and P90G (escinosomes, EL). Optimisation of formulations in terms of vesicle size and sample homogeneity was obtained by the Ultrasonic Homogenizer HD 2200, using the different conditions displayed in Table 1.

### 3.2. Physical Characterization of Nanovesicles

Physical data of the developed nanovesicles are reported in Table 3. All the formulations analyzed by DLS/ELS had sizes ranging from 100 to 140 nm, being CLs the smallest ones. All the nanovesicles had negative ζ-potential values with the highest found for EL. Both CL and EL formulations had low PdI and good deformability, since the ratio of vesicle diameter before and after extrusion was close to the unit (Table 3). ECL did not display deformability. Then, the three different formulations loaded with BRB were also characterized in terms of average diameter, PdI, ζ-potential, and deformability (Table 3). The best homogeneity and deformability parameters were found for EL loaded with BRB (B-EL) and conventional liposomes loaded with BRB (B-CL). The high flexibility of the membrane allow vesicles to pass through skin pores, even smaller than the vesicle diameter, and control the transport of ESN and BRB through the different skin layers. A slight increase in size was observed for B-EL when compared with the unloaded vesicles (Table 3), but this was not expected to affect skin permeability.

**Figure 2:**
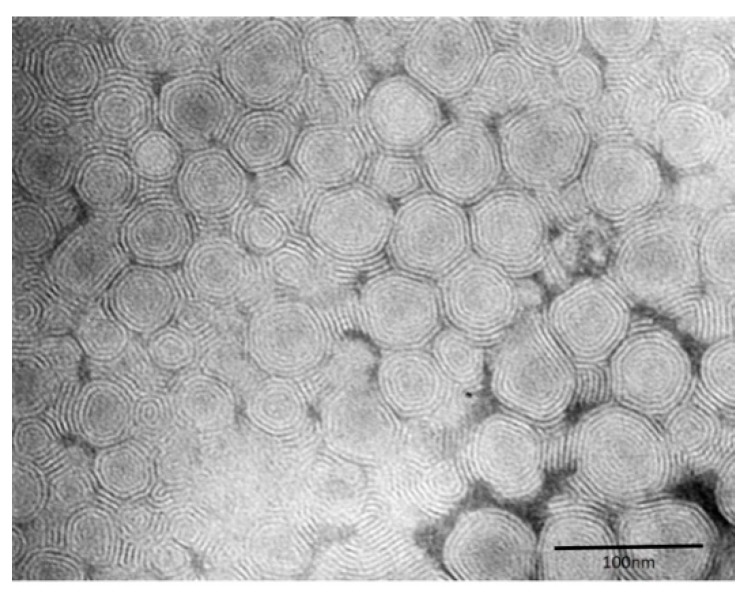
TEM image of empty escinosomes (EL).

**Figure 3:**
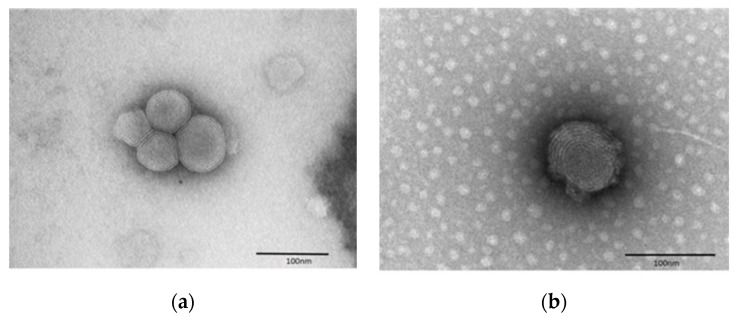
Morphological comparison by TEM between (**a**) empty escinosomes (EL) and (**b**) BRB loaded escinosome (B-EL).

TEM analysis with the negative staining technique was performed to evaluate the morphology and architecture of the nanovesicles, in particular escinosomes (EL, composed only by P90G and ESN, Figure 2). By this procedure, the whole liposomes appeared clearly outlined on an electron-dense background. The vesicles had spherical shape, a membrane-like envelope and often showed an inner lamellar structure (Figure 2). Similarly, after loading escinosomes with BRB, the B-EL formulation (Figure 3b) still maintained the spherical shape and the lamellar structure of empty escinosomes (Figure 3a).

### 3.3. Technological Characterization of Nanovesicles

In Table 4 the chemical characterization of all liposomes is reported and expressed as drug concentration, recovery, and encapsulation efficiency. When 0.5% ESN was added to the vesicles both R% and EE% were more than 95% for both ECL and escinosome (EL) formulations (Table 4). Interestingly, the loading of 0.13% BRB to obtain B-CL, BRB loaded escinosome (B-EL) and B-ECL resulted in a similar R%, but the resulting EE% were quite different (Table 4). EE% of B-CL and B-ECL were quite similar (ca. 44% and 47%, respectively), while EE% of BRB in B-EL was more than 66.7%. The pH was about 5 in all the developed liposome dispersions.

### 3.4. Stability Studies of the Developed Nanocarriers

**Figure 4:**
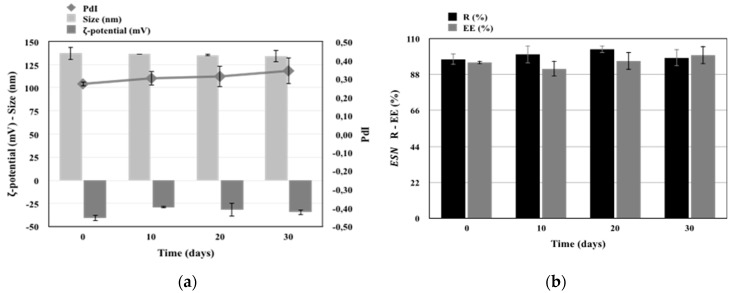
Graphs of the physical and chemical stability studies of EL (escinosome): (**a**) size, ζ-potential and PdI (Polydispersity Index); (**b**) R (Recovery) and EE (Encapsulation Efficiency) of ESN. Data are shown as mean ± SD (*n =* 3).

**Figure 5:**
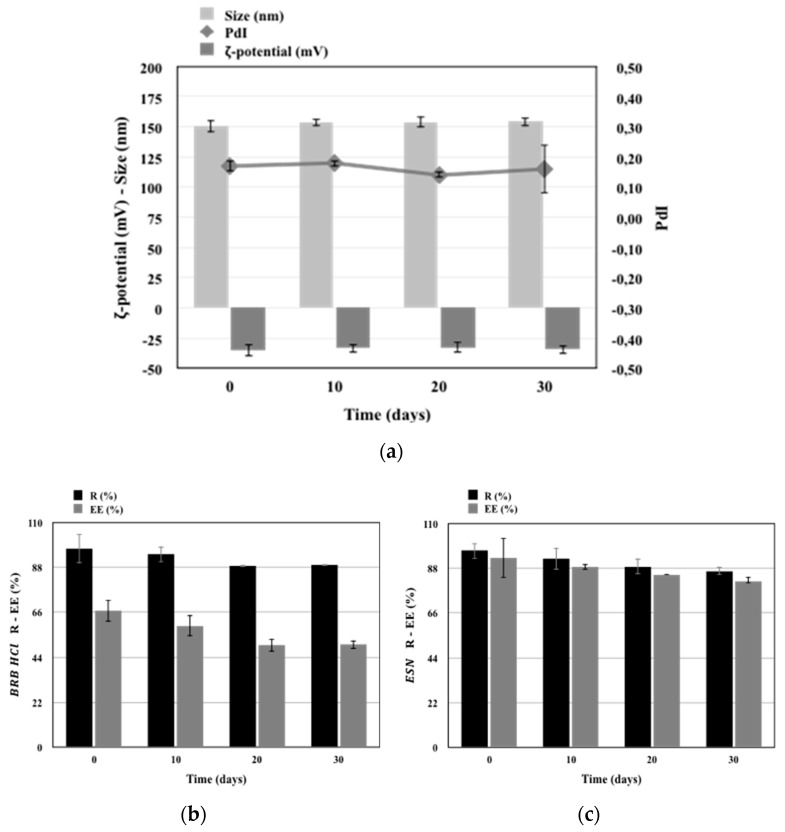
Graphs of the physical and chemical stability studies of B-EL (BRB loaded Escinosome): (**a**) size, ζ-potential, and PdI (Polydispersity Index); (**b**) R (Recovery) % and EE (Encapsulation Efficiency) % of BRB; (**c**) R (Recovery) % and EE (Encapsulation Efficiency) % of ESN. Data are shown as mean ± SD (*n =* 3).

Escinosomes (EL) and BRB loaded escinosomes (B-EL), selected as the two formulations with the most promising chemical and physical characteristics, were tested for stability. Both formulations were kept protected from light at 4 °C and their physical and chemical parameters were monitored every 10 days. EE%, average size, PdI, and ζ-potential were rather stable during the test. Overall, both samples remained stable for up to 1 month under proper storage conditions (Figure 4 and Figure 5). EL physical stability in terms of size, PdI, and ζ-potential is reported in Figure 4a. After 30 days of storage, the ζ-potential value ranged from −40 mV to ca. −35 mV. PdI was very stable during the storage period, while sizes of vesicles increased slightly. EL chemical stability in terms of R% and EE% of ESN was displayed in Figure 4b.

B-EL physical stability in terms of size, PdI, and ζ-potential is reported in Figure 5a, which shows that all values remained constant during the storage period. Figure 5b,c reported the B-EL chemical stability in terms of R% and EE% of loaded BRB and B-EL chemical stability in terms of R% and EE% of loaded ESN respectively. A slight decrease in both R% and EE% of BRB was found after 30 days storage, while ESN remained highly loaded in the vesicles.

### 3.5. Release Studies of ESN and BRB from Vesicles

**Figure 6:**
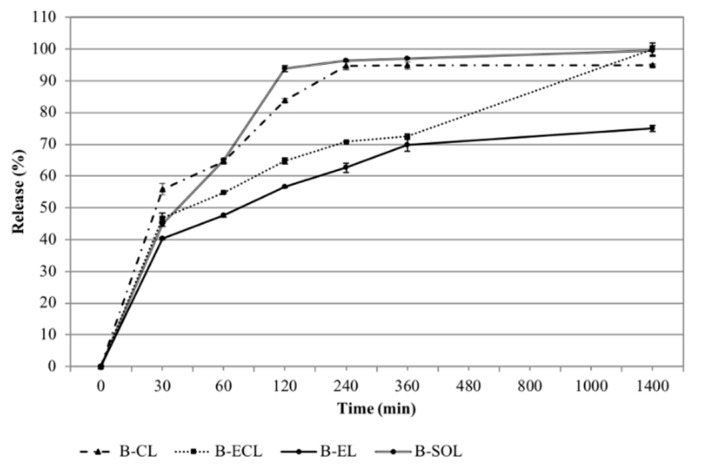
BRB release (%) from B-CL (conventional liposome loaded with BRB), B-ECL (conventional liposome loaded with ESN and BRB), B-EL (BRB loaded escinosome), and B-SOL (BRB aqueous solution). Data are shown as mean ± SD (*n =* 3).

Many methods are reported in the literature to evaluate the release kinetics of vesicles, useful to predict their in -vivo performance. No standard methods and guidelines are established so far. In this study, BRB and ESN release from the different vesicles was investigated by adopting the dialysis bag method, under constant temperature (37 °C) and sink conditions. The release of both BRB and ESN were investigated using the HPLC method reported in the experimental part. B-EL showed the most interesting kinetics (Figure 6). In the first 30 min, only 40% of BRB was released, followed by a prolonged release in the following hours attaining 75% after 24 h. This release kinetic suggests that this formulation could provide a persistent delivery of BRB over time if administered in vivo. B-CL showed a similar release rate, but attaining about 100% of BRB released after 24 h, while B-ECL showed the fastest release kinetic, very close to B-SOL one, with almost 95% of BRB released after 2 h. By contrast, ESN was only detectable after 24 h because of its low UV-visible absorption and low detectability by HPLC-DAD, but also because of its very slow release. In fact, despite a faster release from the aqueous dispersion E-W (22.18 ± 3.66%), ESN reached not more than 18% after 24 h in all formulations. This behavior can be explained by the fact that ESN was strongly retained in the vesicles possibly conferring them a high physical stability. ESN could therefore really represent a vesicle bilayer forming material.

### 3.6. Hyaluronidase Inhibition Assay

In the present study, ESN anti-inflammatory, venotonic, and anti-oedematous potential activities, once it is formulated in B-EL, were evaluated by the hyaluronidase inhibition assay [39,40]. In fact, different pathological processes related to inflammation, venous insufficiency, and oedema present hyaluronidase deficiency. The hyaluronidase inhibition activity of ESN formulated in B-EL (BRB loaded escinosome) was investigated and compared to that of free ESN, as well as the inhibition activity of free BRB and CL (used as control) was evaluated. The assay was carried out using a colorimetric method reported by Murata and coworkers [29]. CL samples did not exhibit any inhibitory effects on hyaluronidase, as estimated, while ESN was found to show inhibitory effects on the enzyme, as previously reported by Facino at al. [39]. To be specific, IC_50_ of free ESN resulted 197 ± 19 μM, while IC_50_ of ESN loaded in B-EL was 210 ± 18 μM. By contrast, free BRB did not exhibit any inhibitory activity at the concentration used for B-EL. Accordingly, ESN inhibitory effect on hyaluronidase was unaffected by the formulation and the presence of BRB.

### 3.7. Skin-PAMPA™ in Vitro Permeation

In order to predict BRB and ESN absorption across the outermost layer of the skin, a simple and fast strategy was applied using the Skin-PAMPA^TM^, a high reproducible fast test to predict trans-cutaneous penetration of compounds. This is a preliminary permeation test where the chemical composition of the synthetic membranes simulates the *stratum corneum*. Piroxicam and progesterone were used as reference with low and high P_e_, respectively. BRB loaded in B-ECL showed a better permeability across the artificial membranes, since the P_e_ was higher when compared with the other formulations (Table 5). The highest P_e_ of BRB found for B-ECL formulation could be explained by the higher lipophilicity of the vesicle due to the simultaneous presence of ESN and CHOL. Meanwhile, the very similar P_e_ of BRB for B-CL and BRB loaded escinosome (B-EL) formulations could further support the hypothesis that ESN, in addition to being a vesicle bilayer forming material, could even have a role in allowing the *stratum corneum* penetration of vesicles, measured in terms of BRB permeated and recovered in the acceptor compartment of the skin-PAMPA^TM^. By contrast, ESN was not detectable in the acceptor compartment, probably because the vesicle structure remained stable during the short tested period (5 h) and ESN could not cross this artificial membrane.

### 3.8. Histological Evaluation of Rabbit Ear Skin and Human Abdominal Skin

**Figure 7:**
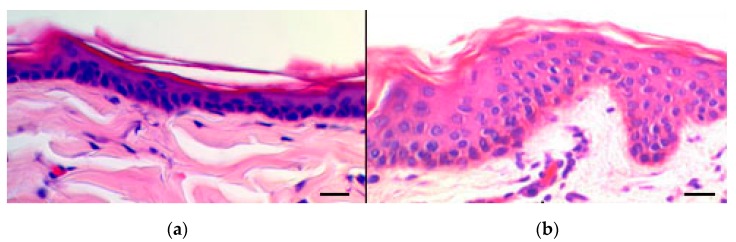
Light microscopic images of (**a**) rabbit ear skin and (**b**) human abdominal skin. In both specimens, the epidermal epithelia show a thin *stratum corneum*. Bars = 50 μm.

The rabbit ear skin was reported to possess a similar thickness of the *stratum corneum* compared with pig ear skin, although the underneath epidermal layers have a slightly different structure, for this reason, it has been proposed as a reliable model for in vitro transdermal tests [35]. In the present study, the rabbit ear skin tissue was histologically evaluated as a substitute for human abdominal skin. Figure 7 shows representative histological sections of rabbit ear skin (Figure 7a) and human abdominal skin (Figure 7b). In both pictures, the various layers of the epidermis and the dermis can be easily identified. To note that, in some areas, the *stratum corneum* appears detached from the underlying layers, probably due to freezing and sectioning artifacts. The histological structure was very similar in both tissues, with the exception of sweat glands, which were lacking in the rabbit skin. The results suggested that the rabbit ear skin is a suitable model to study both lipophilic and hydrophilic permeants.

### 3.9. Percutaneous Penetration Studies and Subsequent Histological Analysis Using Rabbit Ear Skin

The diffusion process through the skin primarily depends on the *stratum corneum*, the main and rate-limiting skin barrier. Since this is not a viable layer, its functionality is similar in vivo and in vitro [41], thereby the in vitro skin permeation model has been largely used over years to predict the human percutaneous absorption, even if it has not yet been validated. In the present study, the rabbit ear skin was used to evaluate the in vivo permeation of the developed nanovesicles.

**Figure 8:**
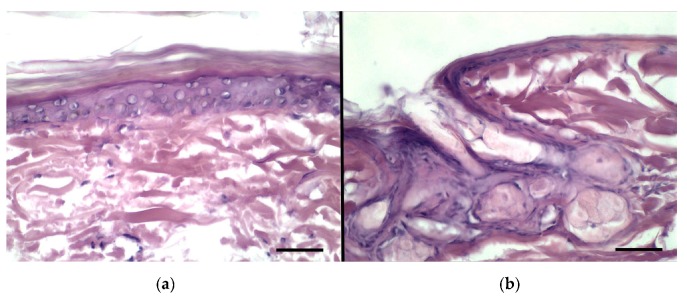
Histological features of rabbit ear skin after the permeation test: (**a**) epidermis, (**b**) hair follicle and sebaceous gland. Bars = 50 μm.

The experiment was performed using the vertical diffusion Franz cells in order to compare the permeation ability of the three different vesicles loaded with BRB inside the skin layers and across the skin, as well as to predict BRB absorption. The permeation study was carried out using the finite dose technique, namely applying doses normally used in clinical conditions. In fact, human exposure to chemicals by topical application of drug is usually of few milligrams of drug per square centimeter of skin, 2–5 mg/cm^2^ [41]. As a first step of our investigation, a conventional histological analysis of the rabbit ear skin samples taken after the permeation studies were carried out (Figure 8). The investigation allowed to exclude the occurrence of major morphological alterations due to the sampling and handling procedures, thus accounting for reliability of the permeation assay.

**Figure 9:**
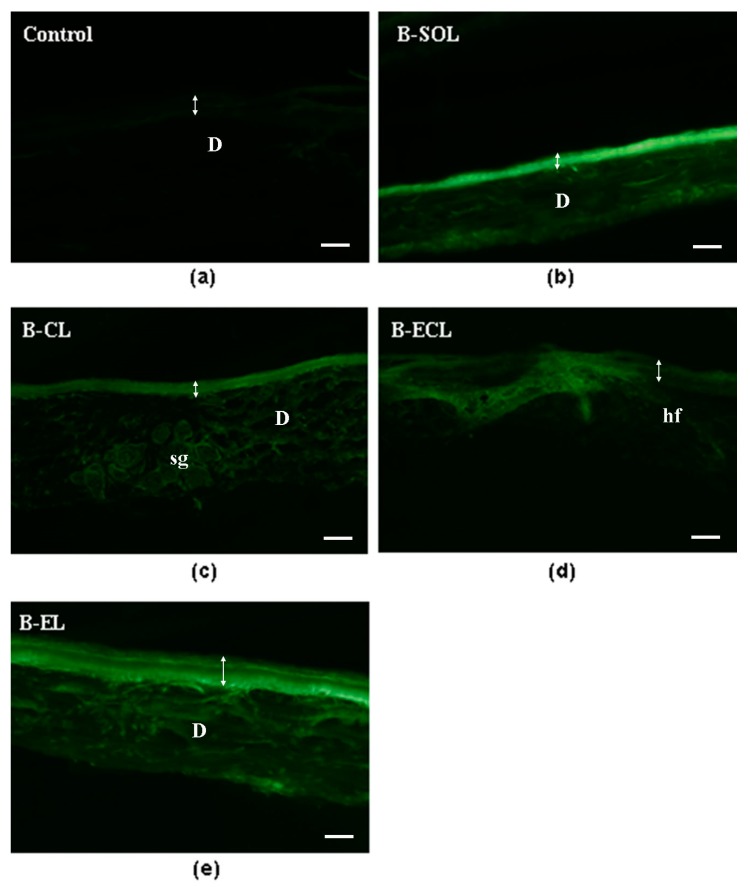
Yellow-green fluorescence images of BRB in sections of rabbit ear skin samples examined after 6 h of permeation test: (**a**) untreated skin (control); (**b**) skin treated with B-SOL (BRB aqueous solution); (**c**) skin treated with B-EL (BRB loaded escinosome); (**d**) skin treated with B-ECL (conventional liposome loaded with ESN and BRB); and (**e**) skin treated with B-CL (conventional liposome loaded with BRB). The experiments were performed in triplicate. D, dermis; sg, sebaceous gland; hf, hair follicle. The double arrow indicates the width of the epidermis. Bars = 50 μm.

**Figure 10:**
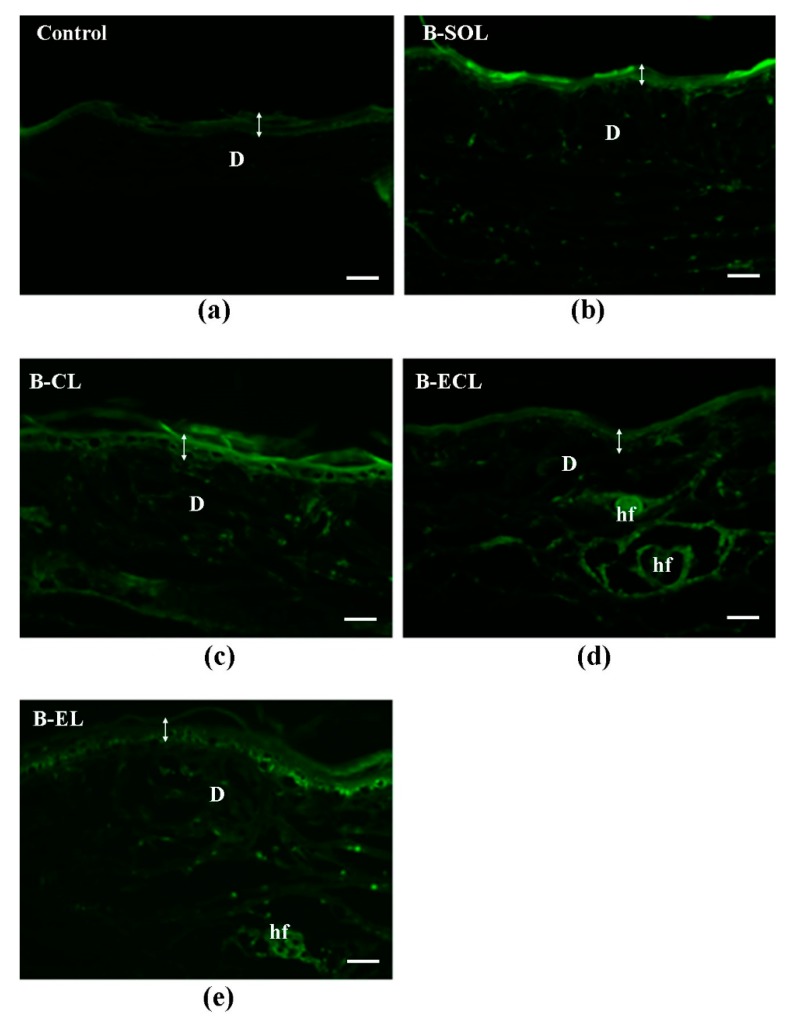
Yellow-green fluorescence images of BRB in sections of rabbit ear skin samples examined after 24 h of permeation test: (**a**) untreated skin (control); (**b**) skin treated with B-SOL (BRB aqueous solution); (**c**) skin treated with B-EL (BRB loaded escinosome); (**d**) skin treated with B-ECL (conventional liposome loaded with ESN and BRB); and (**e**) skin treated with B-CL (conventional liposome loaded with BRB). Experiments were performed in triplicate. D, dermis; hf, hair follicle. The double arrow indicates the width of the epidermis. Bars = 50 μm.

Then, sections of rabbit ear skin samples were examined by UV fluorescence microscopy after 6 h (Figure 9) and 24 h (Figure 10) of permeation. Distribution of BRB in the rabbit ear skin samples was easily evidenced by emission of a specific yellow-green fluorescence under UV excitation. Figure 9 provides the comparison between the untreated rabbit ear skin (Figure 9a), the skin treated with B-SOL (Figure 9b) and the three formulations with BRB (Figure 9c–e) after 6 h of permeation. B-SOL was used as reference of free-BRB permeation. When B-SOL was assayed (Figure 9b), it was evidenced that, after 6 h of permeation test, BRB remained entrapped in the *stratum corneum*, which displayed the highest fluorescence when compared with the other samples (Figure 9c–e). In fact, the permeation test using B-EL and B-ECL (Figure 9c,d) after 6 h showed a weaker florescence of the *stratum corneum* and a more uniform distribution of BRB in the epidermal layers. The 6 h permeation test with B-CL (Figure 9e), on the other hand, displayed a strong florescence of the *stratum corneum*, but also a uniform distribution of BRB from the epidermal layers to the dermal-epidermal junction (DEJ), similarly to B-SOL (Figure 9b).

These findings can be explained by the lower EE% of BRB in B-CL formulation compared to the other formulations (the not entrapped BRB is free to permeate immediately) as well as by the faster BRB release from B-CL than from the other formulations. All these factors result in a greater permeation of BRB loaded in B-CL, in the early hours of the experiment.

After 24 h of permeation test using B-SOL (Figure 10b), BRB florescence was mainly present in the region of DEJ. Only a weak fluorescence remained in the epidermal layers, index of the fast permeation of free-BRB across the skin. By contrast, after the same test period using B-ECL (Figure 10d), BRB still showed the smallest and less uniform permeation.

This behavior can be attributed to the lack of deformability of the vesicles (data are shown in Table 3) and consequently their greater retention in the upper layers of the skin. Meanwhile, the 24 h permeation test with B-CL (Figure 10e), showed a behavior very similar to that of BRB loaded escinosome (B-EL, Figure 10c), with a uniform distribution of BRB fluorescence in the epidermal layers and a good fluorescence to level of the DEJ. Thereby, despite the initial faster permeation of BRB loaded in B-CL than in B-EL, a comparable fluorescence distribution was found after 24 h, index of a comparable ability in allowing BRB absorption. The advantage related to B-EL formulation is the more controlled permeation of BRB, probably explained by the higher EE% of BRB and the slower release of BRB over time (results are shown in Table 4 and Figure 6, respectively). Moreover, the comparable permeability of B-CL and B-EL can also support the initial hypothesis that ESN is a vesicle bilayer forming material and can simulate CHOL as bilayer component of the vesicles, imparting physical stability, as well as allowing a certain deformability and permeability.

To confirm the results obtained by florescence microscopy, the percutaneous penetration of BRB in the rabbit ear skin was successively evaluated by HPLC quantitative analysis of the solutions/samples in the acceptor/donor compartments of Franz cells, as well as of the skin eluates (Table 6). The following permeation parameters were investigated: the absorbed dose after 24 h of permeation (A24), expressed as percentage of the applied dose absorbed through the skin and recovered in the acceptor compartment; the absorbable dose retained inside the skin after 24 h (S24), expressed as percentage of the applied dose recovered inside the skin; and the total absorbed dose after 24 h (TA24), expressed as the sum of A24 and S24. In keeping with the florescence microscopy data, B-SOL (0.13% solution of BRB in water) yielded the highest TA24 value (ca. 1.5%) after application of 1 mL of sample and more than a half of permeated BRB (TA24) was retained above the DEJ (S24), in the skin (ca. 0.9%), as also evidenced in Figure 10b. As expected by fluorescence microscopy, a similar TA24 value (ca. 1%) was found for B-CL. According to Figure 10e and Table 6, the conventional liposomes accumulated in the *stratum corneum* and also reached the epidermal layers beneath, in fact S24 was quite high around 0.8%. However, the percentage of BRB recovered in the receptor compartment was much lesser than that found after the permeation test of B-SOL (Table 6): A24 was close to those obtained with the other liposome formulations, B-ECL and BRB loaded escinosome (B-EL). By contrast, TA24 values after the 24 h of permeation with B-EL and B-ECL were much lower (0.51% and 0.57%, respectively) than that obtained with B-CL (1.11%), by about a half, because only a little amount of BRB was retained in the skin (S24 was ca. 0.2%, Table 6). Overall, A24 found for liposome loaded with BRB was lower than that found for B-SOL and it can be stated that liposomes allowed a slower permeation of BRB than the aqueous solution.

## 4. Discussion

Nanoparticle formulations represent a smart approach to increase stability and/or solubility and as a consequence bioavailability of natural products [23,26,29,42,43,44] but also to enhance permeation and crossing biological barriers including the blood–brain barrier [45,46,47].

Particularly, nanovesicles have great ability to modulate drug penetration through the skin and allow sustained and controlled release of the encapsulated drugs. Their composition, size and charge strongly affect their interaction with the skin and penetrability: therefore, a direct evaluation of the skin penetration enhancement in comparison with conventional drug formulations is helpful for the optimization of efficient nanopharmaceuticals for dermatological purposes. Different in vitro approaches have been suggested to investigate the permeation of drugs loaded in nanovectors as alternative to human skin, principally skin from different animals and, more recently, smart simulation systems such as Skin-PAMPA^™^. These tests can provide a valuable alternative to human skin but in some cases, due to obvious anatomic and physiological differences, they show substantial differences in trans-cutaneous absorption as compared with human skin.

In this study, BRB, a natural quaternary ammonium benzylisoquinoline alkaloid, was chosen as the active drug to be loaded in the nanovesicles, due to the numerous biological activities against diverse skin diseases. This alkaloid is hydrophilic in nature (log P −1.5), and its water solubility is pH- and temperature-dependent. A solution of about 2 mg/mL of BRB was reported to be stable at 25 °C [48].

In this study, three different nanovesicles (CL, ECL, and escinosomes EL), based on P90G, CHOL, and ESN, were developed and optimized. In particular, ESN, a natural mixtures of structurally correlated pentacyclic triterpene saponins [49], largely employed as a vasoprotective anti-inflammatory, anti-edematous and antinociceptive agent, was used at a dose of 5 mg/mL to formulate ECL and EL nanovesicles.

BRB was loaded in the developed nanovesicles and the physical and chemical properties were investigated in terms of AHD, PdI, ζ-potential, deformability, R% and EE%, stability, and release kinetics. The results demonstrated that B-EL possesses the best characteristics for skin application, particularly optimal PdI (0.17) and deformability, high-negative ζ-potential value, great EE (ca. 67%), high physical and chemical stability and the best BRB release properties (ca. 75% after 24 h). At the end of the release study, only 16%–18% of ESN was found in the release medium, indicating that ESN was strongly retained in the vesicles and suggesting that ESN could work as a bilayer-forming material. ESN hyaluronidase inhibition activity was unaffected by the formulation and the presence of BRB. Thus, ESN anti-inflammatory, venotonic and anti-oedematous potential activities were preserved, once it was formulated as vesicle component.

The Effective Permeability (P_e_) of BRB in solution (B-SOL) or loaded in vesicles, namely B-CL, B-ECL, and B-EL was firstly evaluated using the Skin-PAMPA^™^, a smart, validated test for fast prediction of human skin penetration through an artificial membrane mimicking the human *stratum corneum*. P_e_ of B-SOL (1.12 × 10^−6^ cm/s) was very similar to that of progesterone (1.19 × 10^−6^ cm/s), used as reference standard drug of high permeability. Surprisingly, B-ECL displayed a 4-fold higher P_e_ value than that calculated for B-SOL, probably explained by the simultaneous presence of ESN and CHOL. B-CL and B-EL displayed similar P_e_ values, which were twice times the value of B-SOL. These results evidenced that ESN was able to replace CHOL not only structurally but also functionally.

The in vitro permeation studies were preceded by the histological comparison between rabbit ear skin and human abdominal skin. Both tissues showed comparable characteristics with respect to the *stratum corneum* and epidermal thickness as well as hair follicular structure and density. The permeation of BRB with the different formulations was then evaluated by the vertical diffusion Franz cells, observing the distribution of BRB fluorescence and performing quantitative analysis. Despite the higher TA_24_ values found for B-CL, it was found that the skin samples treated with B-EL displayed a comparable BRB absorption as compared with those treated with B-CL. B-EL and B-CL displayed similar composition, except for ESN replacing CHOL in B-EL. CHOL in the bilayer structure of liposomes influences the lipid dynamics because of its rigid ring structure, reducing gauche–trans isomerization and rotational and lateral diffusion of lipids, which results in an ordering effect on the liquid crystalline state with a final fluidizing effect in the gel state [50]. Probably the rigid ring structure of ESN, which is very similar to that of sterols, could have a similar, significant effect on membrane dynamics, confirming that ESN was not simply encapsulated but it is included within the membrane bilayer of the vesicle, giving physical stability and allowing a certain deformability and permeability. It is noteworthy that these two nanovesicles shared major favourable characteristics for skin delivery purposes, i.e., high deformability, proper size, PdI, and ζ-potential. In addition, B-EL was superior to B-CL in terms recovery, encapsulation efficiency, and release properties.

Of note, the results of the permeation tests with rabbit ear skin and Skin-PAMPA™ were substantially different. The latter test principally simulated the permeation through the *stratum corneum*, the main barrier of the epidermis owing to the presence of an abundant lipid matrix containing ceramides, CHOL and free fatty acids, constituents that are different from those of the lower epidermal layers. These characteristics can clarify the faster crossing properties of the epidermis shown by BRB loaded in liposomes when compared with aqueous BRB. By contrast, the test with rabbit ear skin indicated a different performance of the aqueous BRB, with improved permeation through the deepest epidermal layers, *annexa* and dermis, characterized by less lipids and more aqueous tissues. The behavior of BRB in the Skin-PAMPA™ and in the test with rabbit ear skin showed striking differences with the results reported in the literature, which identify BRB having high hydrophilicity and low dermal permeability [20]. Indeed, BRB has an amphiphilic structure, ascribable to the hydrophilic portion of the quaternary ammonium salt and to the lipophilic benzylisoquinoline moiety: these features can extremely influence its penetration through the *stratum corneum,* as well as the underlying epidermal layers. Future studies should be focused on evaluating the concentration role of ESN, when combined with P90G, in the bilayer stability and on better investigating the cohesive interactions occurring between the sugar groups of ESN and the phospholipid headgroups, possibly driven by hydrogen bonds, in particular with the carbonyl and negatively charged phosphate groups of the phospholipid.

## 5. Conclusions

This study highlights that escinosomes and nanovesicular structures composed of P90G and ESN, are stable and deformable vectors able to load BRB, still preserving the anti-hyaluronidase activity of ESN and representing formulation with favorable skin penetration features for the development of new efficacious dermatological vectors. The presence of two active molecules (ESN and BRB), in the developed escinosomes, represents an interesting and promising tool, with possible synergistic activity between ESN and the loaded drug.

## Figures and Tables

**Table 1:** Optimization of CL (conventional liposome), ECL (conventional liposome loaded with ESN) and EL (escinosome) in terms of AHD and PdI, by sonication. Data are shown as mean ± SD (*n =* 3).

Formulation	Composition (Ratio *w/w*)	Sonication Time (s)/Sonication Cycles	AHD (nm)	PdI
CL	P90G-CHOL (33:10)	150 s	106.2 ± 8.1	0.24 ± 0.01
ECL	ESN-P90G-CHOL (5:33:10)	60 s	180.7 ± 3.2	0.40 ± 0.01
ECL	ESN-P90G-CHOL (5:33:10)	150 s	152.6 ± 9.1	0.39 ± 0.03
ECL	ESN-P90G-CHOL (5:33:10)	300 s/5 cycles	142.9 ± 3.1	0.38 ± 0.02
ECL	ESN-P90G-CHOL (5:33:10)	300 s	132.4 ± 0.5	0.36 ± 0.01
ECL	ESN-P90G-CHOL (5:33:10)	600 s/5 cycles	127.3 ± 6.2	0.38 ± 0.03
EL	ESN-P90G (5:33)	30 s	139.1 ± 1.2	0.27 ± 0.01
EL	ESN-P90G (5:33)	60 s/5 cycles	137.4 ± 6.9	0.26 ± 0.01
EL	ESN-P90G (5:33)	60 s	103.2 ± 2.3	0.34 ± 0.04
EL	ESN-P90G (5:33)	150 s	104.1 ± 11.5	0.41 ± 0.03
EL	ESN-P90G (5:33)	180 s	107.9 ± 2.9	0.43 ± 0.01
EL	ESN-P90G (5:33)	300 s	117.9 ± 12.1	0.40 ± 0.02
EL	ESN-P90G (5:33)	600 s/5 cycles	70.0 ± 1.4	0.40 ± 0.01

CL, ECL, and EL showed the best PdI in the following conditions: 150 s of sonication, 10 min of sonication for 5 cycles and 60 s of sonication for 5 cycles, respectively. Thereby, these formulations were selected and loaded with BRB. The formulations with BRB were similarly optimized by the Ultrasonic Homogenizer HD 2200 using different sonication times, as reported in Table 2. B-CL, B-ECL, and B-EL formulations showed the lowest PdI after 10 min of sonication for 5 cycles and they were selected for the following studies.

**Table 2:** Optimization of B-CL (conventional liposome loaded with BRB), B-ECL (conventional liposome loaded with ESN and BRB) and B-EL (BRB loaded escinosome) in terms of AHD (Average Hydrodynamic diameter) and PdI (Polydispersity Index), by sonication. Data are shown as mean ± SD (*n =* 3).

Formulation	Composition (Ratio *w/w*)	Sonication Time (s)/Sonication Cycles	AHD (nm)	PdI
B-CL	BRB+P90G-CHOL (1.3:33:10)	150 s	164.0 ± 25.7	0.28 ± 0.04
B-CL	BRB+P90G-CHOL (1.3:33:10)	300 s/5cycles	124.4 ± 7.6	0.31 ± 0.06
B-CL	BRB+P90G-CHOL (1.3:33:10)	600 s/5 cycles	106.5 ± 18.1	0.26 ± 0.02
B-ECL	BRB+ESN-P90G-CHOL (1.3:5:33:10)	150 s	178.5 ± 9.6	0.25 ± 0.02
B-ECL	BRB+ESN-P90G-CHOL (1.3:5:33:10)	180 s	187.8 ± 1.9	0.23 ± 0.03
B-ECL	BRB+ESN-P90G-CHOL (1.3:5:33:10)	300 s/5 cycles	178.0 ± 6.3	0.23 ± 0.00
B-ECL	BRB+ESN-P90G-CHOL (1.3:5:33:10)	300 s	191.7 ± 1.6	0.22 ± 0.02
B-ECL	BRB+ESN-P90G-CHOL (1.3:5:33:10)	600 s/5 cycles	166.1 ± 23.2	0.20 ± 0.02
B-EL	BRB+ESN-P90G (1.3:5:33)	150 s	238.9 ± 79.7	0.26 ± 0.05
B-EL	BRB+ESN-P90G (1.3:5:33)	180 s	195.9 ± 2.8	0.22 ± 0.02
B-EL	BRB+ESN-P90G (1.3:5:33)	300 s/5 cycles	227.0 ± 76.7	0.23 ± 0.05
B-EL	BRB+ESN-P90G (1.3:5:33)	300 s	275.6 ± 20.9	0.26 ± 0.04
B-EL	BRB+ESN-P90G (1.3:5:33)	600 s/5 cycles	150.1 ± 5.2	0.17 ± 0.02

**Table 3:** Physical characterization of all liposomes.

Formulation	Composition (Ratio *w/w*)	AHD (nm)	PdI	ζ-Potential (mV)	Deformability
CL	P90G-CHOL (33:10)	106.2 ± 8.1	0.24 ± 0.01	−26.9 ± 1.3	1.05 ± 0.04
ECL	ESN-P90G-CHOL (5:33:10)	127.3 ± 6.2	0.38 ± 0.03	−31.4 ± 2.5	*
EL	ESN-P90G (5:33)	137.4 ± 6.9	0.26 ± 0.01	−40.5 ± 3.1	0.89 ± 0.01
B-CL	BRB + P90G-CHOL (1.3:33:10)	106.5 ± 18.1	0.26 ± 0.02	−29.5 ± 0.2	1.02 ± 0.01
B-ECL	BRB + ESN-P90G-CHOL (1.3:5:33:10)	166.1 ± 23.2	0.22 ± 0.02	−30.1 ± 0.4	*
B-EL	BRB + ESN-P90G (1.3:5:33)	150.1 ± 5.2	0.17 ± 0.02	−34.8 ± 5.1	1.09 ± 0.01

AHD = Average Hydrodynamic Diameter; PdI = Polydispersity Index; * = not deformable. CL = conventional liposome; ECL = conventional liposome loaded with ESN; EL = escinosome; B-CL = conventional liposome loaded with BRB; B-ECL = conventional liposome loaded with ESN and BRB; B-EL = BRB loaded escinosome. Data are shown as mean ± SD (*n =* 3).

**Table 4:** Chemical characterization of all liposomes. R = Recovery; EE = Encapsulation Efficiency. CL = conventional liposome; ECL = conventional liposome loaded with ESN; EL = escinosome; B-CL = conventional liposome loaded with BRB; B-ECL = conventional liposome loaded with ESN and BRB; B-EL = BRB loaded escinosome. Data are shown as mean ± SD (*n =* 3).

Formulation	ESN			BRB		
	ESN% *w/v*	R%	EE%	BRB% *w/v*	R%	EE%
CL	/	/	/	/	/	/
E-CL	0.5%	98.04 ± 0.12	97.14 ± 0.72	/	/	/
EL	0.5%	97.34 ± 3.40	95.46 ± 0.93	/	/	/
B-CL	/	/	/	0.13%	95.76 ± 2.58	44.10 ± 0.71
B-ECL	0.5%	98.02 ± 0.23	94.80 ± 0.11	0.13%	96.21 ± 0.32	47.60 ± 0.56
B-EL	0.5%	96.50 ± 3.90	93.16 ± 9.68	0.13%	97.06 ± 7.10	66.70 ± 5.33

**Table 5:** Effective Permeability (P_e_) of BRB loaded in B-CL (conventional liposome loaded with BRB), B-ECL (conventional liposome loaded with ESN and BRB), B-EL (BRB loaded escinosome), and B-SOL (BRB aqueous solution) and of the standard references (piroxicam and progesterone). Results are shown as mean ± SD (*n =* 3).

Formulation	P_e_ (10^−6^ cm/s)
B-CL	2.06 ± 0.01
B-ECL	4.30 ± 1.87
B-EL	2.16 ±0.02
B-SOL	1.12 ± 0.03
Piroxicam	0.24 ± 0.10
Progesterone	1.19 ± 0.35

**Table 6:** Permeation parameters: A_24_ (Absorbed dose), S_24_ (absorbable dose retained inside the Skin), and TA_24_ (Total Absorbed dose) related to B-SOL (BRB aqueous solution), B-EL (BRB loaded Escinosome), B-ECL (Conventional Liposome loaded with ESN and BRB), and B-CL (Conventional Liposome loaded with BRB). Results are shown as mean ± SD (*n* = 3).

Formulation	BRB		
	A_24_ (%)	S_24_ (%)	TA_24_ (%)
B-SOL	0.60 ± 0.37	0.92 ± 0.17	1.52 ± 0.05
B-EL	0.32 ± 0.01	0.19 ± 0.06	0.51 ± 0.03
B-ECL	0.33 ± 0.03	0.24 ± 0.04	0.57 ± 0.06
B-CL	0.32 ± 0.13	0.79 ± 0.11	1.11 ± 0.04
